# Neuronal correlates of voluntary facial movements

**DOI:** 10.3389/fnhum.2015.00598

**Published:** 2015-10-28

**Authors:** Martin Krippl, Ahmed A. Karim, André Brechmann

**Affiliations:** ^1^Department of Methodology, Psychodiagnostics and Evaluation Research, Institute of Psychology, Otto-von-Guericke University MagdeburgMagdeburg, Germany; ^2^Department of Psychiatry and Psychotherapy, Universitätsklinikum TübingenTübingen, Germany; ^3^Department of Prevention and Health Psychology, SRH Fernhochschule RiedlingenRiedlingen, Germany; ^4^Special Lab Non-Invasive Brain Imaging, Leibniz Institute for NeurobiologyMagdeburg, Germany

**Keywords:** somatotopy, voluntary facial movements, FACS, neural correlates, fMRI

## Abstract

Whereas the somatotopy of finger movements has been extensively studied with neuroimaging, the neural foundations of facial movements remain elusive. Therefore, we systematically studied the neuronal correlates of voluntary facial movements using the Facial Action Coding System (FACS, Ekman et al., 2002). The facial movements performed in the MRI scanner were defined as Action Units (AUs) and were controlled by a certified FACS coder. The main goal of the study was to investigate the detailed somatotopy of the facial primary motor area (facial M1). Eighteen participants were asked to produce the following four facial movements in the fMRI scanner: AU1+2 (brow raiser), AU4 (brow lowerer), AU12 (lip corner puller) and AU24 (lip presser), each in alternation with a resting phase. Our facial movement task induced generally high activation in brain motor areas (e.g., M1, premotor cortex, supplementary motor area, putamen), as well as in the thalamus, insula, and visual cortex. BOLD activations revealed overlapping representations for the four facial movements. However, within the activated facial M1 areas, we could find distinct peak activities in the left and right hemisphere supporting a rough somatotopic upper to lower face organization within the right facial M1 area, and a somatotopic organization within the right M1 upper face part. In both hemispheres, the order was an inverse somatotopy within the lower face representations. In contrast to the right hemisphere, in the left hemisphere the representation of AU4 was more lateral and anterior compared to the rest of the facial movements. Our findings support the notion of a partial somatotopic order within the M1 face area confirming the “like attracts like” principle (Donoghue et al., 1992). AUs which are often used together or are similar are located close to each other in the motor cortex.

## Introduction

The face is the window to our feelings, emotions and therefore important in our interpersonal relationships. Understanding how facial movements are controlled is therefore an important way to understand what happens in our brain during the expression of emotions or the communication of social signals. Additionally it is crucial for treating neuropsychiatric disorders associated with lack of facial movements in social interaction (Krippl and Karim, 2011; Yoshimura et al., 2015). Since Jackson (1873) postulated specific neural correlates to different movements, several studies have tried to investigate the neural foundations of body movements. Penfield and Rasmussen (1950) found for instance a somatotopic order of the human body in the sensorimotor primary cortex. However, a somatotopic order within the facial area has not been demonstrated yet.

The study of somatotopy in humans had its prominent beginning with Penfield and Boldrey (1937) and Penfield and Rasmussen (1950). They detected the well-known “homunculus” in the primary motor cortex (M1) and the primary sensory cortex. However, there are some constraints of this work which need to be considered. First, there is evidence according to Lemon (2008) and others (Leyton and Sherrington, 1917, cited according Woolsey et al., 1952; Donoghue et al., 1992; Rathelot and Strick, 2006; Lemon, 2008) that instead of a strict somatotopy a better description of M1 organization is to speak of “multiple and overlapping representation of movement” (Lemon, 2008, p. 801). He states that “this type of representation is well suited for the many and varied combination of ‘fractional’ movements into useful actions” (Lemon, 2008, p. 801). Ejaz et al. (2015) showed in agreement with the latter findings that the frequency of combined muscle usage can predict the structure of representations in the motor cortex. However, this does not exclude the possibility that each muscle or AU could have a hot spot where it is accentuated, and these hot spots could be arranged in a strict somatotopic order (Meier et al., 2008).

Second, although the homunculus suggests that there is also an orderly representation of facial muscles along the superior-inferior body axis, Penfield and Rasmussen (1950) did not describe specific muscles of the face, but only “movement of face” or “brow.” Their description of lip movements was also not differentiated into different types of lip movements. Third, Penfield’s studies involved epileptic patients, so it is not clear if healthy people have the same anatomical and functional structure in the motor cortex as epileptic patients. Additionally, the stimulating electrodes were typically placed on lateral portions of the cortex and not deep within the sulci.

Fine grained somatotopic organization has only been studied in the domain of finger movements (Kleinschmidt et al., 1997; Dechent and Frahm, 2003). Although movement of different fingers revealed a vast overlap of activation in M1, subtracting the different fingers yielded a systematic somatotopic representation. Dechent and Frahm (2003, p. 282) concluded that “The human M1 hand area presents as a physiologically synergetic and anatomically interconnected area, with fine-scale somatotopy implemented as a quantitative predominance of individual digit representations sharing common areas.”

Whereas the somatotopy of finger movements has been extensively studied with neuroimaging, the neural foundations of facial movements remain elusive.

A considerable part of the research on facial movements comes from lesion patients. Singh et al. (2011) investigated a patient with intact spontaneous facial movements but loss of voluntary control of facial movements caused by a bilateral insular infarct. Remarkably, Sim et al. (2005) showed an inverse dissociation during a bilateral insular infarct, meaning loss of emotional facial movements but preservation of voluntary facial movements.

Rinn (1984) described the differences in the neural background of voluntary and spontaneous facial movements as differences related to pyramidal and extrapyramidal systems. Hopf et al. (1992) support this distinction based on the observation that lesion patients can have either emotional or volitional paresis while still being able to perform facial movements the other way. Both state that voluntary facial movements need an intact motor cortex, whereas spontaneous/emotional facial movements are controlled by the extrapyramidal system with the most important center in the basal ganglia. Gazzaniga and Smylie (1990) examined three split brain patients and showed that both hemispheres can produce spontaneous facial movements, but only the left hemisphere is efficient in producing voluntary expressions. Based on all these and their own findings, Ross et al. (2007) set up a model of neuronal control during primary emotions vs. volitional facial movements and hypothesized that faces are organized on the upper-lower facial axis. This hypothesis is based on the studies of Ross et al. (2007) showing that facial blends (mixtures of emotions) are more easily and accurately posed on the upper-lower than on the right-left hemiface. Ross and Pulusu (2013) assume that primary emotions evolve from the right hemisphere, whereas social emotions and display rules are controlled by the left hemisphere. In support of this hypothesis, Ross and Pulusu (2013) showed that voluntary smiles began in the majority of cases on the right side of the face, while spontaneous facial expressions began mostly on the left side of the face.

Pizzamiglio et al. (1987) compared unilaterally brain damaged persons using the “Requested Facial Action test” in which a film shows one actor performing several movements (including many movements from the upper and lower face). The participants then had to imitate the observed movements. Using this task Pizzamiglio et al. (1987) found that patients with right brain damage were significantly more impaired in imitating several Action Units (AU; upper and lower face) than persons with left brain damage. The right hemisphere seems to be more involved in specific facial movements. Remarkably, the size of the lesion was not correlated to the impairment of the movement.

Ogura et al. (2011) investigated oral movements in healthy participants as used in dysphagia rehabilitation and demonstrated increased brain activity in the precentral gyrus and the cerebellum. Martin et al. (2004) compared tongue movements with swallowing, showing higher activity during tongue movements in many regions (left lateral pericentral and anterior parietal cortex, anterior cingulate cortex (ACC), supplementary motor area (SMA). Precuneus, rostral ACC, and insula have been shown as preferentially activated in swallowing compared to tongue movements. Grabski et al. (2012) asked participants to perform lip, jaw, larynx, and tongue movements. The four conditions activated a set of largely overlapping, common brain areas (sensorimotor and premotor cortices, the right inferior frontal gyrus, the SMA, the left parietal operculum, the adjacent inferior parietal lobule, the basal ganglia, and the cerebellum). In an imaging study, Meier et al. (2008) found a facial somatotopy from dorsal to lateral for right side squinting, lip (pursing forward and drawing back) and tongue movements (forward and backward).

Moreover, there are two studies specifically related to facial movements associated to emotions. Iwase et al. (2002) investigated voluntary smiling and found higher regional cerebral blood flow in the face area of the left M1 and bilateral SMA than during laughter/smile induced by visual comics. Hanakawa et al. (2005) instructed participants to unilaterally move the risorius muscle or fingers. They found overlapping regions of activity for hand and face movements in the ipsilateral BA6 on the precentral gyrus. On the contralateral side, face activity was localized ventrolateral to the “hand-knob” portion of the precentral gyrus.

Although Iwase et al. (2002) and Hanakawa et al. (2005) studied facial movements related to emotions, they did not refer to the AU concept based on the Facial Action Coding System (FACS; Ekman et al., 2002), which is a central concept in the current study. AUs are simultaneous movements of one, two or more muscles and can be seen as the smallest units of facial movements. The FACS describes 46 AUs in an objective way, meaning that only visually detectable characteristics of facial movements are described. There is no included interpretation of AUs as emotions like in other facial coding systems (Restle and Brown, 1970). AUs are generally viewed as a more important than muscle units, because Ekman and Friesen (1978) judged differentiations between movements of a specific muscle and a combination of movements of several muscles to be unreliable in some cases. Another reason was that it allows for the separation of “more than one action from what most anatomists described as one muscle” (Ekman et al., 2002, p. 5). Therefore, it is plausible to assume that instead of individual muscles, different AUs are topographically represented in the motor cortex. This has already been stated by other researchers, although not related to the FACS (e.g., Leyton and Sherrington, 1917, cited in Lemon, 2008). In this article we define AUs as movements which are exactly defined in the FACS.

According to Morecraft et al. (2001, 2004), the upper face in the rhesus monkey is controlled by M2 but not by M1. Several researchers have adopted this view also regarding human research (e.g., Cattaneo and Pavesi, 2014). However, Penfield and Rasmussen (1950) have shown that the “brow” (no more detail of the type of movement was described) can be moved by stimulating M1 in human patients. Additionally, other studies have demonstrated activity in M1 during movement of the upper face (e.g., Meier et al., 2008).

There is, however, no study explicitly comparing the representation of different AUs in the face according to FACS. Therefore, the main goal of the current study was to examine whether different AUs indeed show different activation patterns in M1. Participants in this study were trained in producing the AUs by a certified FACS coder (first author). We also analyzed other brain areas that have been suggested to be somatotopically organized [premotor cortex (PMC), SMA, putamen, and insula; Maillard et al., 2000; Indovina and Sanes, 2001; Schubotz and von Cramon, 2003; Brooks et al., 2005; Arienzo et al., 2006].

A further goal was to investigate whether the activity peaks of the AUs would show a topographical pattern from superior to inferior facial muscles. We hypothesized that the movement of the frontalis muscle (AU1 + 2) elicits the highest BOLD response, superior to the other movements, followed by activity of the corrugator muscle (AU4), the zygomaticus (AU12), and finally by the orbicularis oris (AU24). To analyze the activation patterns, peak activity within each region of interest (ROI) was determined. FMRI data were also analyzed using a whole brain analysis to reveal which areas all over the brain were active during facial movements.

## Material and Methods

### Participants

Eighteen participants (9 men; *M* = 24.22 years, *SD* = 2.37) were selected from an original group of 27 participants who performed the MRI study. Nine participants were excluded from analyses because they were unable to perform the required facial movements without co-activating additional AUs^1^. The exclusion was based on FACS codings of the facial movements in the MRI scanner, which were grabbed by a DVD recorder from the camera signal. Most of the problems occurred with AU4, where some participants often additionally performed AU7 (lid tightener). Some participants had also problems with AU1 + 2 and additionally performed AU5 (upper lid raiser). Inter-individual differences could be the reason for these participants’ ability to perform specific AUs well and others not. We do not know what differences this might be. Therefore, we wanted to prevent a bias from coming into the data through these participants and hence excluded them from all direct mapping analyses and not only from those where, their performance was very bad.

One of the participants was only excluded from differential mapping, leaving 17 participants. All participants gave written informed consent to the study, which was approved by the ethics committee of the University of Magdeburg.

### Movements

Four different facial movements were selected: eye brow raising (AU1+2), brow lowering (AU4), lip corner pulling (AU12) and lip pressing (AU24). The selection of movements was made to include movements from the upper face and the lower face and with an eye to which movements were relatively easy to perform. Additionally, the selected movements are associated with negative emotions (AU4; AU24) as well as positive emotions (AU12).

#### Training

The participants were trained to perform different AUs and AU combinations in a separate session 2–6 days before the scanner session. Participants were instructed to perform the facial movements as strongly as possible but to avoid other movements. If they had problems preventing other movements, they were instructed to reduce the intensity of the intended AU. This instruction aimed to elicit the highest selectivity and amplitude of fMRI activation for the respective AUs, which is true when the movement is performed with the highest possible intensity (signal), but only when the relevant movement is not mixed with any others (noise). Observation of the movements done in the scanner was also done by a certified FACS coder (first author).

### Design

Two types of runs were performed (adapted from Dechent and Frahm, 2003), because they have been successfully used in other studies on somatotopy: the first type (direct mapping) contained one movement (AU, with a duration of 12 s) alternated with a pause (18 s). The second type of run (differential mapping) consisted in two alternating movements (each 12 s). We had four direct mappings (one for each movement) and six differential movements (one for each combination of the movements).

In the movement phases, a movement had to be repeated every 2 s. Therefore, the sign which showed the participants that they had to move (a green point or a red point) blinked in a rhythm of 2 s beginning 2 s after onset.

We did not intentionally manipulate the complexity of the movements in our study, although some movements may be more complex in the sense that more muscles are moved at once compared to other movements. During direct mapping, we used only repetitions and no alternations of movements. During differential mapping, there was one alternation every 12 s. Thus, the level of complexity regarding this aspect of the design was minimal for the direct mapping task and a little higher for the differential mapping task.

### MRI

The study was performed in a 3-Tesla Scanner (Siemens Trio, Erlangen) with an open head coil on which a camera was mounted for control of the facial movements. T1-weighted MPRAGE sequences for the anatomy (192 slices of 1 mm each) and EPI sequences (TR = 2000 ms, flip angle = 80°, *FOV* = 192 mm, matrix = 64^∗^64) for the functional data were collected. The resolution of the functional data was 3^∗^3^∗^3 mm (33 slices, 0.3 mm gap, *TE* = 30 ms).

During scanning, participants wore earplugs and their head was fixed with a cushion. In the fMRI Session, stimuli (a red or a green point demarcating when the movements had to be performed) were displayed using the Presentation software^2^ run on a standard PC computer and back-projected onto a screen which could be viewed via a mirror mounted on the head coil. Participants’ faces were observed and recorded using an eye-tracking camera prepared for usage in the MRI-scanner (Kanowski et al., 2007). Data were analyzed with BrainVoyagerQX1.9 using a boxcar function shifted by 4 s to account for hemodynamic latencies (Bandettini et al., 1993; Dechent and Frahm, 2003). Spatial realignment correction for head motion was used as implemented in BrainVoyager. Normalization was performed using co-registration of EPI images and anatomical images. No smoothing was applied, Estimated translation and rotation parameters were inspected and never exceeded 3 mm translation and 2° rotation (Goebel et al., 2006). Therefore, no subject was excluded due to movement artifacts.

### Peak Activity Determination

To determine the peak activity in each ROI, we used Talairach coordinates to decide whether an active voxel was within one of the following areas: M1-BA4, premotor BA6, Putamen, nucleus caudatus, insula, or somatosensory cortex. A voxel was attributed to a region if it was up to two mm away (according to the Talairach daemon, Talairach and Tournoux, 1988). With Brainvoyager, the *T*-value level of visualized voxels was raised step by step until only one voxel within a ROI was shown. The talairach daemon was used to indicate whether a voxel was within the relevant ROI (e.g., M1). For the computation of the ROI-GLMs the ROIs were defined as three by three mm cubes with the peak as the center-voxel.

## Results

### Whole Brain Analyses of Direct Mapping

**FIGURE 1:**
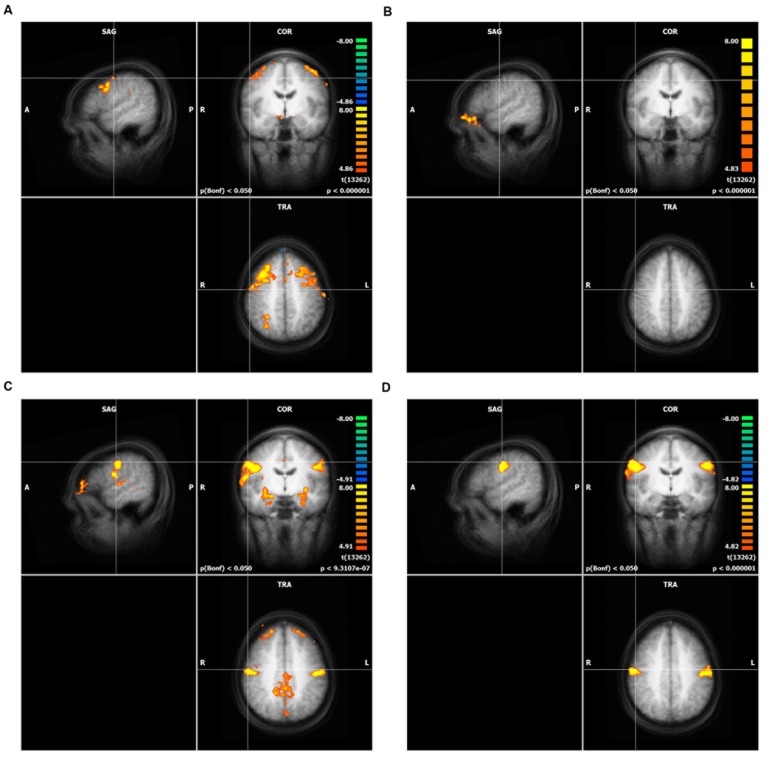
**Result of the whole brain analysis of direct mapping for each action unit (AU), **(A)** AU1+2, **(B)** AU4, **(C)** AU12, **(D)** AU24**. The crosshair in each of the four panels is located at the peak activity. Only clusters with more than 100 voxels are shown. The significance level was cut off at *T*-values lower than 8.0 (see heat map). Intensity threshold 1, **(A)** AU1+2, **(B)** AU4, **(C)** AU12, **(D)** AU24.

All four movement conditions showed highly significant activations (Bonferroni corrected *p* < 0.01) compared to the resting condition in the following brain areas (bilaterally if not stated differently, **Figure 1**): M1, premotor cortex (PMC), SMA, insula, somatosensory cortex (BA1, 2, 3), putamen, cerebellum (anterior lobe and posterior lobe, only the dorsal part was measured), thalamus, dorsal ACC, BA44, BA22, BA43, right BA40, and visual cortex (BA17, 18, and 19). Visual cortex areas were probably activated because the subjects had to look at the screen and detect whether the shown fixation-point was red or green. The cerebellum is also a well-known motor region. However, it was not in the focus of interest of the current study.

### ROI-Analyses of Direct Mapping

Since brain activation by facial movements can be expected to overlap to some degree, a differential mapping approach is not necessarily suitable for revealing small shifts in peak activation. Therefore, we used direct mapping to identify the most significant foci of activation for each movement condition in M1, and also in PMC, SMA, and putamen. The results revealed different peak areas for the four AUs in nearly all motor areas except the right and the left PMC, where AU1+2 and AU4 showed the same peak and the left putamen, where AU12 and AU24 revealed the same peak coordinates (**Table 1**).

**Table 1:** ROI-GLM for different brain areas.

	ROI-Center-Coordinates Talairach (MNI in parenthesis) of Peak activities			ROI-GLM (relevant AU against the mean of the other AUs)			
	*x*	*y*	*z*	Beta AU1+2	Beta AU4	Beta AU12	Beta AU24
Right M1 AU1+2	51 (51.5152)	-7 (-9.5776)	46 (49.5572)	0.622	0.481^∗∗^	0.560	0.415^∗∗∗^
Left M1 AU1+2	-45 (-45.4545)	-10 (-12.9757)	52 (55.9078)	0.642^∗∗∗^	0.311^∗∗∗^	0.341^∗∗∗^	0.318^∗∗∗^
Right PMA AU1+2	42 (42.4242)	-7 (-9.7322)	49 (52.814)	*0.633*^∗∗∗^	0.575	0.536	0.385^∗∗∗^
Left PMA AU1+2	-45 (-45.4545)	-7 (-9.7322)	49 (52.814)	*0.616*^∗∗∗^	0.503^∗^	0.436^∗∗∗^	0.475^∗∗^
SMA AU1+2	-3 (-3.0303)	-7 (-9.8868)	52 (56.0708)	0.924^∗∗∗^	0.780^∗∗^	0.737^∗∗∗^	0.785^∗∗^
Right Putamen AU1+2	24 (24.2424)	-1 (-1.3903)	7 (7.5449)	0.516	0.574	0.622°	0.485
Left Putamen AU1+2	-24 (-24.2424)	-4 (-4.6338)	10 (10.6387)	0.566	0.597	0.605	0.490
Right Insula AU1+2	51 (51.5152)	-31 (-32.8978)	19 (18.9423)	0.589^∗^	0.493	0.598	0.368^∗∗∗^
Left Insula AU1+2	-48 (-48.4848)	-28 (-29.8089)	19 (19.1053)	0.428^∗∗^	0.256^∗∗^	0.336	0.192^∗∗∗^
Right M1 AU4	51 (51.5152)	-7 (-9.423)	43 (46.3004)	0.625°	0.493	0.545	0.329^∗∗^
Left M1 AU4	-51 (-51.5152)	-4 (-6.3341)	43 (46.4634)	0.625°	0.493	0.545	0.329^∗∗^
Right PMA AU4	42 (42.4242)	-7 (-9.7322)	49 (52.814)	0.633	*0.575*^∗^	0.536	0.385^∗∗∗^
Left PMA AU4	-45 (-45.4545)	-7 (-9.7322)	49 (52.814)	0.616°	*0.503*	0.436	0.475
SMA AU4	-1 (-1.0101)	-4 (-6.7979)	52 (56.2338)	0.888	0.889	0.827	0.868
Right Putamen AU4	24 (24.2424)	2 (1.6986)	7 (7.7078)	0.408^∗∗∗^	0.615^∗∗^	0.581	0.436^∗∗∗^
Left Putamen AU4	-21 (-21.2121)	-1 (-1.3903)	7 (7.5449)	0.409^∗∗∗^	0.625^∗∗^	0.587	0.484^∗∗^
Right Insula AU4	48 (48.4848)	-31 (-33.0524)	22 (22.1991)	0.354^∗∗∗^	0.548^∗∗∗^	0.454	0.314^∗∗∗^
Left Insula AU4	-48 (-48.4848)	-37 (-39.0756)	19 (18.6163)	0.273	0.311	0.318	0.169^∗∗^
Right M1 AU12	54 (54.5455)	-10 (-12.2028)	37 (39.6239)	0.366^∗∗∗^	0.265^∗∗∗^	0.869^∗∗∗^	0.853
Left M1 AU12	-48 (-48.4848)	-13 (-15.4463)	40 (42.7177)	0.358^∗∗∗^	0.273^∗∗∗^	0.823^∗∗∗^	0.690^∗^
Right PMA AU12	48 (48.4848)	-13 (-15.1372)	34 (36.2041)	0.288^∗∗∗^	0.257^∗∗∗^	0.945^∗∗∗^	0.921
Left PMA AU12	-54 (-54.5455)	-4 (-5.7158)	31 (33.4362)	0.470^∗^	0.213^∗∗∗^	0.584^∗∗∗^	0.599
SMA AU12	-3 (-3.0303)	-4 (-6.6433)	49 (52.977)	0.860	0.777	0.835	0.756
Right putamen AU12	24 (24.2424)	2 (1.8532)	4 (4.451)	0.388^∗∗∗^	0.597	0.629^∗∗∗^	0.424^∗∗∗^
Left Putamen AU12	-24 (-24.2424)	-1 (-1.3903)	7 (7.5449)	0.409^∗∗^	0.625^∗^	*0.587*^∗∗^	0.484^∗∗^
Right Insula AU12	51 (51.5152)	-31 (-32.8978)	19 (18.9423)	0.589	0.493	0.598^∗∗^	0.368^∗∗∗^
Left Insula AU12	-54 (-54.5455)	-37 (-39.2302)	22 (21.8731)	0.195^∗∗∗^	0.227^∗∗^	0.381^∗∗∗^	0.204^∗∗^
Right M1 AU24	51 (51.5152)	-10 (-12.2028)	37 (39.6239)	0.467^∗∗∗^	0.360^∗∗∗^	0.847	0.874^∗∗∗^
Left M1 AU24	-54 (-54.5455)	-13 (-15.4463)	40 (42.7177)	0.325^∗∗∗^	0.334^∗∗∗^	0.696	0.734^∗∗∗^
Right PMA AU24	45 (45.4545)	-7 (-9.7322)	49 (52.814)	0.456	0.426	0.493	0.403
Left PMA AU24	-51 (-51.5152)	-4 (-5.7158)	31 (33.4362)	0.448^∗∗∗^	0.160^∗∗∗^	0.527	0.623^∗∗∗^
SMA AU24	0 (0)	-4 (-6.7979)	52 (56.2338)	0.863	0.839	0.776	0.829
Right Putamen AU24	24 (24.2424)	-1 (-1.0812)	1 (1.0313)	0.255^∗∗∗^	0.559	0.535	0.522
Left Putamen AU24	-24 (-24.2424)	-1 (-1.3903)	7 (7.5449)	0.409	0.625	0.587^∘∘^	*0.484*
Right Insula AU24	51 (51.5152)	-31 (-32.8978)	19 (18.9423)	0.589^∘∘∘^	0.493°	0.598^∘∘∘^	0.368^∘∘∘^
Left Insula AU24	-51 (-51.5152)	-37 (-39.0756)	19 (18.6163)	0.269	0.094^∗∗^	0.205	0.258

*Stars in the right four columns represent significant contrasts of AUs mentioned in the head of these columns compared with the AUs mentioned in the left column (with brain areas). When the AU in the left column is the same as in the head of the four right columns, the number represents the overall contrast of the AU against the other AUs. In case different AUs have the same peak-coordinates, the relevant AU was tested against all other AUs but not against the AU with the same peak-coordinates. This case was marked by italics*.

*If, as in case of the right insula, three AUs have the same peak-coordinates, the overall significances are reported as usual. Tests with stars conform with the hypotheses: relevant AU higher than others. ^∗^*p* < 0.05, ^∗∗^*p* < 0.01, ^∗∗∗^*p* < 0.001. Closed circles represent significant results in the opposite direction: relevant AU lower than others. °*p* < 0.05, ^∘∘^*p* < 0.01, ^∘∘∘^*p* < .001*.

*For transformation of Talairach to MNI coordinates we used the program tal2icbm_spm.m, available on the Internet (http://brainmap.org/icbm2tal/index.html)*.

**FIGURE 2:**
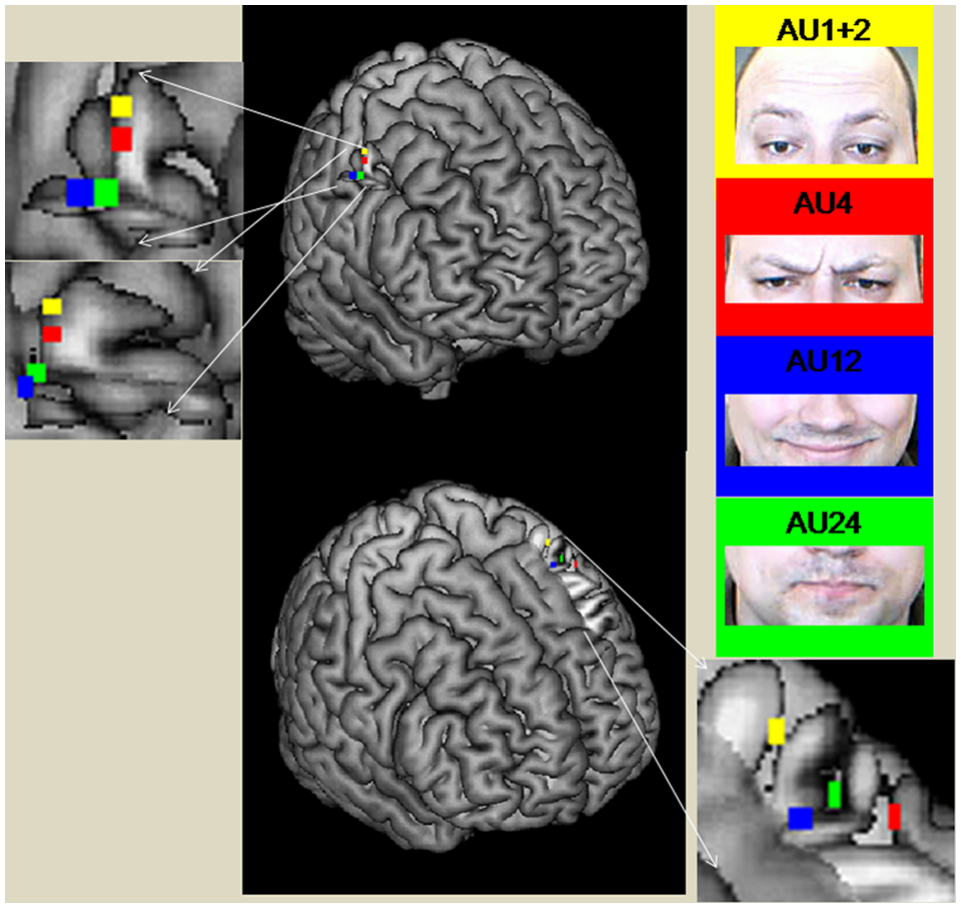
**Peak coordinates of M1 areas during the performance of the different AUs (direct mapping)**. The figure was created with the software Mricron (https://www.nitrc.org/projects/mricron).

In the *right M1*, the coordinates of the peak activities systematically shifted from medial to lateral and from superior to inferior for AU1+2, AU4, AU24, and AU12, with AU12 and AU24 having the same level of inferiority (see **Figure 2**). The peaks of AU12 and AU24 were more posterior than the peaks of AU1+2 and AU4. In *left M1*, the coordinates of the peak activities did not show a systematic shift as was the case in the right M1. We found that the coordinates of the peaks of AU12 and AU24 were very close to each other, whereas that of AU1+2 was more superior and the peak of AU4 more anterior.

In the *right PMC*, the coordinates of the peaks for AU1+2 and AU4 were very close and that for AU24 was also close by. However, the ROI peak for AU12 was far more ventral (**Table 1**).

The *left PMC* showed equal peak coordinates for AU1+2 and AU4. This peak was much more superior and more medial than the peaks for AU24 and AU12. These two peaks were very close to each other. AU24 was more medial than AU12.

The *SMA* showed the highest activations all over the brain. The peaks of all conditions were in the left hemisphere and were very close to each other. The peaks of AU4 and AU24 were overlapping. The peak of AU12 was more inferior and the peak of AU1+2 was more posterior than the other peaks.

The *right putamen* showed different peaks for all movements. The peaks of the movements were all very close to each other. Only AU24 showed more inferior and slightly more posterior peak activity. The *left putamen* revealed the same peak for AU12 and AU24. This peak was more lateral but very close to the peak of AU4. The peak of AU1+2 was a little more posterior and more superior to AU12/24.

Peak activities in the *right insula* were nearly all at one coordinate. The only AU different from the others was AU4, being a little more medial and superior. In the *left insula*, peak activities were more diversely distributed, with AU1+2 being the only movement clearly anterior to the other three, which were close to each other.

### ROI-GLM

ROI-GLM was calculated for each peak ROI to test whether the BOLD response amplitude (beta values) for a given movement was higher than for the other movements (contrasting one AU against the mean of the other three AUs). The results supported such a pattern in many cases, but not in all. Especially, but not exclusively, the ROIs of AU4 often had lower activity than other movements. This is probably due to the fact that AU4 elicited lower overall activity than the other AUs.

### Whole Brain Analyses of Differential Mapping

**FIGURE 3:**
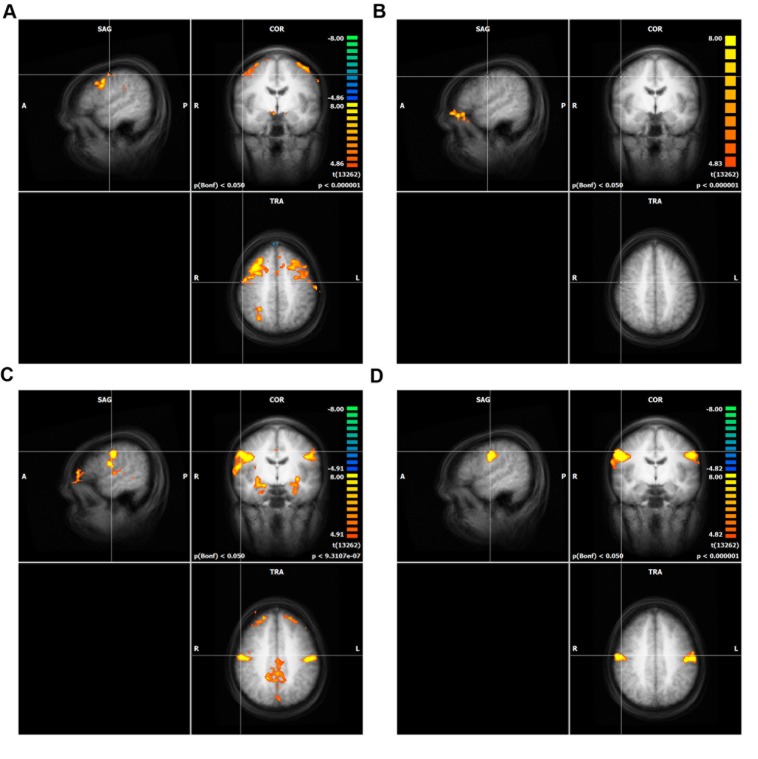
**Whole brain analysis of differential mapping, GLM in Conjunction with experimental condition against the baseline (including resting state)**. The crosshair in each of the four panels is located at the peak activity of direct mapping. Only clusters with more than 100 voxels are shown. The significance level was cut off at Bonferroni *p* = 0.05. Intensity threshold 1, **(A)** AU1+2, **(B)** AU4, **(C)** AU12, **(D)** AU24.

In differential mapping, each AU was compared to the mean of all other AUs. The analysis revealed that the activity of AU4 was smaller than for the other two movements across the entire brain. The result is a not visible activation pattern for this AU (see **Figure 3**). **Figure 3** depicts a conjunction analyses as implemented in BrainVoyager QX (Goebel et al., 2006). A contrast of AU1+2 (or AU12) against the other three movements was combined with the contrast of AU1+2 (or AU12) against the baseline (mean of all conditions, including resting state).

## Discussion

### Whole Brain Analyses

The results of the direct mapping approach showed strong activation throughout all movement conditions in the motor regions of interest, i.e., the M1 areas, PMCs, SMAs, and putamen. This suggests that large parts of these motor areas are enlisted in the production of voluntary movements. Are all of these activated regions necessary to perform the specific facial movements? In Penfield and Rasmussen’s (1950) experiments they were not. The electrical stimulation of only a small region of the motor cortex was sufficient to produce a specific movement. Nevertheless, neuroimaging studies (see Schieber, 1999, p. 941) have shown that “a given muscle is controlled by a large territory in M1 and that the territories for different muscles overlap.” It could be that this large overlap is based on a rudimentary evolutionary connection between facial muscle movements, as described by Ertekin et al. (2013). However, there are distinct peak activities in M1 for each movement evident for facial movements in our data and for finger movements in the data of Dechent and Frahm (2003).

The results of the differential mapping could not contribute much to answering our initial questions because of the fact that overall activity differed strongly between movements. We contrasted each of the conditions against the other three, but only AU1+2 led to a significantly stronger BOLD activity than the other movements. This was an unexpected result because Dechent and Frahm (2003) showed better results for differential than for direct mapping in their finger somatotopy study. The reason for this finding may be that facial muscle control is more interconnected than finger muscle control and thus results in a larger overlap of BOLD activity.

### Peak Activities of Direct Mapping

Peak activations of the different movement conditions were found at different coordinates in most of the motor areas. The relative location of the peaks in M1 corresponds with Penfield and Rasmussen’s (1950) findings roughly, but not in detail. In *the right M1*, the coordinates of the peak activities systematically shifted from medial to lateral for AU1+2, AU4, AU24, and AU12. With the exception that AU24 is not the most lateral peak, this supports Penfield’s facial somatotopy. The peaks of AU12 and AU24 were more posterior than the peaks of the other two AUs. This is against the detailed homunculus assumption; instead, we have an inverse somatotopic order within both lower face areas of M1. Looking at the other AU representations in the *left M1* area, we found that whereas the peak of AU1+2 is more superior to AU12 and AU24 as it was in the right hemisphere, that of AU4 is more anterior and lateral than all of the other AUs. Hence, in the *left* M1 the peak for AU4 is not between AU1+2 and AU12, as assumed by Penfield, but rather anterior to AU12 and AU24. All in all, the findings suggest only a partial somatotopic order of main representations of AUs within the M1 face areas. At least one other principle of organization looks to be influencing the order. The principle of “like attracts like” (Woolsey et al., 1952; Donoghue et al., 1992; Nude et al., 1996) would be a good candidate for such a principle. AUs which are often used together (Ejaz et al., 2015) or are similar are located close to each other in the motor cortex.

We also analyzed the topography of peak activities in the PMA, SMA, and putamen, where similar principles of organization might work as in the right and left M1 area. Specific for the PMA is that the peaks of AU1+2 and AU4 are at the same coordinate for both hemispheres, indicating a need for a study with higher resolution. The peaks for AU12 and AU24 are very close to each other, as in M1. However, as in the M1 area, we also found substantial differences in the topography of peak activities of the movements between both hemispheres within the PMA. In conclusion, the topography of the M1 and PMA areas are roughly similar. In contrast to this finding, the topographies of the SMA and putamen are very different to both M1 and PMA. Peak activities for the SMA and the putamen are very close together. Whereas the SMA and right putamen have a very similar topography, peaks in the left putamen differ substantially from the other two regions. All in all, the resolution used in this study is not sufficient to capture differences between movements within the putamen and SMA.

It is also interesting to compare our peak activities – especially that of AU24 – to Hanakawa et al. (2005) peak activities of activation of the risorius muscle, which could be categorized as AU20 in the FACS (Ekman and Friesen, 1978). Their peak activities according to the Talairach-Daemon^3^ are located in the precentral gyrus and thus in BA 6 (PMC) and not in BA 4 (M1). The coordinates of Hanakawa’s right peak activities of AU20 in the precentral gyrus (BA 6) are clearly more anterior and a little more lateral than the M1 peak of our AU24. Compared to our right PMC peak of AU24, Hanakawa’s peak of AU20 lies more anterior, ventral and medial. The left peak of Hanakawa’s AU20 in the precentral gyrus (BA 6) lies more anterior to our AU 24 M1 peak. Compared to our premotor peak of left AU24, it lies more superior and a little more posterior. As the risorius muscle is located close to the orbicularis oris muscle (AU24) on the ventral-dorsal axis, where it sets the pull at the lower part of the orbicularis oris, our results support the assumption of a somatotopic organization in conjunction with the “like attracts like” principle in the human PMC.

The only brain region we investigated for peak activities which is not a motor area was the insula, known as a somatovisceral area (Davidson and Irwin, 1999). Peak activities in the right insula were nearly all at one coordinate. The only AU different from the others was AU4, being a little more medial and superior. In the left insula, peak activities were more diversely distributed, the AU1+2 being the only movement clearly anterior to the other three, which were close to each other. Therefore, the resolution of our 3-Tesla study was probably too low to detect all of the relevant differences in the insulae.

### Unexpected Results in ROI-GLMs of Direct Mapping

As additional analyses we computed ROI-GLMs to test whether the relevant AUs had the highest BOLD activity of all AUs at the peak coordinates. This would indicate additional support for the somatotopic organization of the studied brain areas, although a significant difference for a higher activation in an AUs peak voxel is not surprising. However, in some of the ROI-GLMs, the relevant AU did not elicit the highest activations. This was mostly the case for AU 4. The reason is probably the vast activation overlap between the different AUs in combination with the fact that AU4 induced generally weaker activation in the motor areas than the other AUs (AU1+2 showed the highest activity in M1 and in the PMC). Only in areas with specificity for one movement did we obtain a significant ROI-GLM result. This effect does not change if only very good performers are analyzed, meaning the intensities of the movements rated by FACS coding are very similar. The results suggest that the intensity of the movement is a potential reason for generally lower AU4 activity in the motor cortex. Participants had difficulties inhibiting other AUs while performing AU4. Thus, they were told to reduce the intensity of the movement to avoid other AUs like AU7. Unfortunately, intensity differences cannot be detected by FACS coding to allow a covariance analysis of the respective BOLD activation, because FACS ratings do not represent interval but only ordinal level. FACS differentiates intensity into five categories, where E represents the maximum category, reflecting the most extreme possible movement, and D reflects a slightly lower intensity. Often it is not possible to reliably differentiate between these intensities with FACS coding. Nevertheless, FACS coding allowed us to conclude that AU4 was performed with less intensity than the other AUs and AU1+2 was performed with strongest intensity. Of course, these ideas are *post hoc* explanations which will require confirmation in future studies on the relation between movement intensity and BOLD activity.

In contrast to intensity, some other researchers might ask whether differences in movement complexity might have led to our results, as it is one of the important parameters influencing activity patterns in the motor areas. Harrington et al. (2000) define the complexity of movements according to their surface structure and their abstract structure. With surface structure, they mean “perceptual or motoric properties of sequences such as the number of movements or the types of effectors” (Harrington et al., 2000, p. 57). Furthermore, the sequence-specific structure is manifested in relations between movements, such as repetitions or alternations (Restle and Brown, 1970; Povel and Collard, 1982; Rosenbaum et al., 1984; Harrington et al., 2000, p. 57). Several studies have shown that the complexity of movements has an effect on the dissemination and the intensity of brain activation (Binder and Rao, 1994; Harrington et al., 2000; Lotze et al., 2000). Hence, this could be an important point to be considered in the interpretation of our results, although we did not manipulate the complexity of movements intentionally.

We used only repetitions and no alternations of movements, and the number of repetitions was equal for all movements. Therefore, complexity was minimal for this sub-characteristic of complexity. However, it may be that one movement is more complex or difficult to perform than another due to other reasons, like the number of involved muscles. In AU4, three muscles (depressor glabellae; depressor supercilli; corrugator supercilii) are involved; in the other AUs only one muscle (zygomaticus major and orbicularis oris) or two (AU1+2 frontalis medialis and lateralis) come into play. Thus, the number of involved muscles cannot explain why AU4 elicits less BOLD activity. The orbicularis oris muscle can be moved in many different ways, and AU24 may thus be most difficult to be reliably produced. However, AU24 did not induce significantly stronger BOLD activity in M1 than the other AUs (with the exception of AU4). Instead, AU1+2 induced the highest activity. Therefore, we believe that the intensity with which the movements were performed and not the complexity of the movements is the most probable explanation for the different levels of overall activity.

A limitation of our results is that they are restricted to participants who are able to perform the relevant AUs without any other AU. Subjects who were not able to perform the relevant AUs without any other AU were excluded. Future studies could probe the neural correlates of AUs in such subjects and investigate the difference between subjects who are able to perform the relevant AUs without any other AU and subjects who are not able to perform this task.

## Conclusion

This is the first study to investigate the neuronal correlates of voluntary facial movements against each other using the FACS (Ekman and Friesen, 1978). The movements performed in the MRI scanner were defined as AU – the smallest units of facial movements – and rated by a certified FACS coder (first author).

Our facial movement tasks generally induced high activations in motor areas (e.g., M1, SMA, PMC, putamen) and the insula. Like finger movement control, facial movement control is organized in “physiologically synergetic and anatomically interconnected areas” (Dechent and Frahm, 2003). However, within regions with overlapping representations (Lemon, 2008), we found distinct peak activities in the left and right M1, supporting a distinction between upper and lower face representation in the right M1 area and a somatotopic organization within the right upper face area of M1 part. Additionally, we found the same order within the lower face representation, according to AU12 and AU24, but in the inverse somatotopic order. Thus, the only distinction between the left and right order of peak activities goes back to AU4, in which the left side is more lateral and frontal not only compared to AU1+2 but even compared to AU24 and AU12. Our findings support the notion of a partial somatotopic order which is in line with the “like attracts like” principle.

Further research should investigate whether and how activation in M1 and other motor regions changes with varying intensity of facial movement.

## Conflict of Interest Statement

The authors declare that the research was conducted in the absence of any commercial or financial relationships that could be construed as a potential conflict of interest.

## Footnotes

1 The excluded participants were mostly participants, which had initial problems to do the movements in the pure form (without any other AU). Thus, they were asked to train their ability to do the movements at home, if they had time, and we conducted the experiment with them, in the hope that they could perform the movements in the right way. But often they were not able to.

2 http://www.neurobs.com

3 http://www.talairach.org/daemon.html
